# Therapeutic Potential and Safety of the *Cinnamomum zeylanicum* Methanolic Extract Against Chronic *Toxoplasma gondii* Infection in Mice

**DOI:** 10.3389/fcimb.2022.900046

**Published:** 2022-06-09

**Authors:** Abdullah D. Alanazi, Hamdan I. Almohammed

**Affiliations:** ^1^ Departmentof Biological Sciences, Faculty of Science and Humanities, Shaqra University, Ad-Dawadimi, Saudi Arabia; ^2^ Department of Basic Science, Faculty of Medicine, Almaarefa University, Riyadh, Saudi Arabia

**Keywords:** toxoplasmosis, *in vivo*, cytokines, toxicity, antioxidant, oxidative stress

## Abstract

**Background:**

This experimental study determined the *in vitro*, *in vivo*, and toxicity effects of *Cinnamomum zeylanicum* methanolic extract (CZME) against *Toxoplasma gondii* infection.

**Methods:**

The *in vitro* activity of CZME *T. gondii* tachyzoites was studied by the MTT (3-(4,5-dimethylthiazol-2-yl)-2,5-diphenyltetrazolium bromide) assay. Infected mice were treated with CZME for two weeks at doses of 20, 40, and 60 mg/kg/day. Then, the therapeutic effects of CZME were evaluated by assessing the mean number and mean size of *T. gondii* tissue cysts, oxidant-antioxidant enzymes, pro-inflammatory cytokines, and mRNA expression levels of bradyzoite surface antigen 1 (BAG1) by real-time PCR.

**Results:**

CZME significantly (p <0.001) increased the mortality rate of parasites in a dose- and time-dependent response. The mean number of intracellular tachyzoites was significantly reduced after CZME therapy. The treatment of infected mice with CZME resulted in a significant (p <0.001) downregulation of BAG1 and the level of lipid peroxidation (LPO) and nitric oxide (NO) as oxidative stress markers. However, a considerable rise (p <0.05) was found in the levels of antioxidant markers such as glutathione peroxidase (GPx), catalase enzyme (CAT), and superoxide dismutase enzyme activity (SOD). In a dose-dependent response, after treatment of infected mice with CZME, the level of pro-inflammatory cytokines of IFN-γ, IL-1β, and IL-12 was considerably elevated. CZME had no significant cytotoxicity on Vero cells, with a 50% cytotoxic concentration of 169.5 ± 5.66 μg/ml.

**Conclusion:**

The findings confirmed the promising therapeutic effects of CZME on chronic toxoplasmosis in mice. Nevertheless, further investigations must confirm these results, elucidate its precise mechanisms, and examine its effectiveness in human volunteers.

## Introduction

Toxoplasmosis is an infectious parasitic disease between humans and animals with a global spread caused by the *Toxoplasma gondii* protozoan (Harrell and Carvounis, 2014). Cats are definitive hosts, and humans, as accidental hosts, can become infected by eating *T. gondii* cysts in raw or uncooked meat, fruits, vegetables, and drinking water contaminated with oocysts (Harrell and Carvounis, 2014). Congenital transfusion, blood transfusion, and organ transplantation are other transmission routes (Saadatnia and Golkar, 2012). Human toxoplasmosis can be described in acquired, congenital, and morbid forms in people with weakened immune systems. Acquired infection occurs in immunocompetent or healthy people, principally as gentle and lymphadenopathy, and seldom results in brain or ocular involvement (Hampton, 2015). In immunocompromised people, however, infection is an important opportunistic disease that can cause encephalitis in these patients (Wang et al., 2017). If toxoplasmosis occurs during pregnancy (congenital infection), it may cause many clinical signs, ranging from asymptomatic cases to abortion and severe brain and ocular involvement (Jones et al., 2001).

Today, pyrimethamine and sulfadiazine plus folate are used as the first choices for the prevention and management of toxoplasmosis in clinics. Other medications such as atovaquone, azithromycin, spiramycin, clarithromycin, dapsone, and cotrimoxazole are sometimes used to treat toxoplasmosis (Harrell and Carvounis, 2014; Ben-Harari et al., 2017). Studies show that despite having potent inhibitory activity on *Toxoplasma*, these chemical anti-*Toxoplasma* agents have side effects such as increased risk of suppression of bone marrow function, hematologic toxicity, teratogenic effects, and limited renal complications (Iaccheri et al., 2008). Despite significant advances in pharmacological and safety research, we still lack the ideal and proper agent for treating chronic toxoplasmosis. Moreover, effective and efficient vaccines have not been commercially introduced for widespread use in humans. Therefore, the development of a new drug with properties such as effectiveness in penetrating the placenta, non-toxicity, and parasitic effects on all stages of the parasite, especially the cystic form, seems necessary.

Recently, several laboratory studies in various parts of the world have reported the anti-*Toxoplasma* effects of some plant extracts and fractions, e.g., *Feijoa sellowiana*, *Eryngium caucasicum*, *Quercus castaneifolia*, *Allium paradoxum*, *Zea mays*, *Eucalyptus methanolic*, *Annona muricata*, *Aloe vera*, and *Sambucus nigra* (Daryani et al., 2015; Ebrahimzadeh et al., 2017; Mirzaalizadeh et al., 2018; Ahmadpour et al., 2019; Miranda et al., 2021). However, due to some limitations, their results have not yet been finalized and are unreliable. Many herbal teas have been used as natural remedies for many diseases for hundreds of years; modern science confirms some of their uses and has found new benefits for them (Poswal et al., 2019). *Cinnamomum zeylanicum* L., or cinnamon (Lauraceae family), also locally called “kurfah,” is a tropical plant that grows in various parts of the world, such as Sri Lanka, India, Indochina, and Madagascar (Ranasinghe et al., 2013). Multiple parts of this plant, especially its inner bark, have various pharmacological and health benefits, e.g., antioxidant, antihypertensive, anti-aging, anticancer, hepatoprotective, immunomodulatory, and antimicrobial effects (Shah and Panchal, 2010; Rahayu et al., 2021). A vital mechanism that plays an essential role in the prevention and even control of toxoplasmosis is the reinforcement of the immune system, especially cellular immunity, by increasing some inflammatory factors and mediators (Sasai et al., 2018). Due to having certain compounds, medicinal herbs have recently displayed promising *in vitro* and *in vivo* anti-*Toxoplasma* and anti-*T. gondii* effects with immunomodulation of animals and host cells (Teixeira et al., 2020; Shaapan et al., 2021).

This experimental study aimed to evaluate the *in vitro, in vivo*, and toxicity effects of *C. zeylanicum* methanolic extract (CZME) against *T. gondii* infection.

## Materials and Methods

### Plant Material

*C. zeylanicum* barks of the plant were obtained from a spice shop in Riyadh, Saudi Arabia, and identified by a botanist at the Department of Basic Science. A voucher sample was engaged at Department of Basic Science, Almaarefa University, Saudi Arabia (No. HA-01R-64).

### Preparing the Methanolic Extract

Dried and powdered bark (200 g) *via* the percolation procedure were extracted with 70% methanol successively for 72 h at room temperature. The solution was then passed through a Whatman filter paper and concentrated at 55°C under vacuum conditions using a rotary evaporator (Heidolph, Schwabach, Germany). The lyophilized extract was preserved at −20°C until testing (Albalawi, 2021).

### Phytochemical Examination

We analyzed CZME for the presence of phytochemicals such as terpenoids, flavonoids, saponins, alkaloids, and tannins using some qualitative experiments explained previously (Albalawi, 2021).

### Total Phenolic Content

The total phenol concentration existing in CZME was identified based on the Folin–Ciocalteu reagent (FCR) technique, as explained by other researchers (Singleton et al., 1999). Briefly, 0.5 of ml CZME (1 mg/ml) was added to 10% FCR (1.5 ml) for 10 min. The mixture was mixed with a 7.5% Na_2_CO_3_ solution and then incubated at 30°C for 2 h. Subsequently, the absorbance of the mixture was measured at 760 nm. The total phenol concentration was reported versus the gallic acid standard as mg/g gallic acid equivalent (GAE) of the dry weight of the extract (mg GAE/g DW).

### Total Flavonoid Content

With the aluminum chloride solution technique, we detected the total flavonoid concentration in CZME as formerly described (Phuyal et al., 2020). In brief, after mixing specific amounts of CZME, distilled water, potassium acetate, and 10% AlCl3, and incubating the mixture for 30 min, the optical absorption of the resulting mixture was read and recorded at 420 nm. Finally, the flavonoid concentration was identified versus a catechin standard and presented as mg/g catechin equivalents of the dry weight of the individual extract (mg CAE/g DW).

### Gas Chromatography/Mass Spectrometry (GC/MS) Analysis

The chemical structure of CZME was determined using a Trace GC-ISQ mass spectrometer (Thermo Scientific, Austin, TX, USA) supplemented by a capillary HP-5MS column (30 m × 0.25 mm, 0.25 mm film thickness). The temperature of the column was initiated at 56°C, set to 185°C at a rate of 5°C/min, and continued at 260°C for 5 min. The temperatures of the injector and the interface were 230 and 235°C, respectively. The helium flow rate was 1 ml/min with a 70 eV ionization voltage. The compositions were identified through the evaluation of the retention times and mass spectra with those of the WILEY 09 and NIST 11 mass spectral databases (Adams, 2004; NIST, N., 2014; Alyousif et al., 2021).

### Parasite

The parasite *T. gondii* avirulent strain of ME49 was provided by Shaqra University, Saudi Arabia, and was prepared by intraperitoneal injection into BALB/c mice.

### Cell Culture

To study the *in vitro* anti-*Toxoplasma* evaluation of CZME, Vero cells were procured from American Type Culture Collection (No. CCL-81) and maintained in the RPMI-1640 medium enhanced with 10% inactivated fetal bovine serum (FBS), 100 units/ml of penicillin, and 100 μg/ml of streptomycin, and kept at 37°C with 5% CO_2_.

### *In Vitro* Anti-Toxoplasma Effects

Some concentrations of CZME (25, 50, and 100 µg/ml) were separated into the wells containing *T. gondii* tachyzoites (1 × 10^6^ cells/ml) for 30–180 min at 37°C. Next, MTT powder (3-(4,5-dimethylthiazol-2-yl)-2,5-diphenyltetrazolium bromide) (5 mg/ml) was added to the tested wells and were against incubated under the same conditions. After adding the stop solution (dimethyl sulfoxide), the optical density (OD) of the wells was read at 570 nm using an ELISA reader (LX800; Biotec, USA) (Alnomasy, 2021). Normal saline-treated tachyzoites and tachyzoites treated with atovaquone were considered the negative and positive controls, respectively.

### Effect on Infection Rate and Intracellular Parasites

The activity of CZME on the rate of infection and tachyzoites was as follows: after seeding the Vero cells (1 × 10^5^ cells/ml) in the 24-well plate and keeping them warm at 37°C for one day, they were infected with *T. gondii* tachyzoites (1 × 10^6^/ml). After one day, the infected Vero cells were incubated with CZME at concentrations of 12.5 to 100 µg/ml for 3 h. Finally, after staining the treated cells with Giemsa, microscopic assessment was carried out to study the infection rate and the mean number of intracellular parasites by checking 100 cells (Alnomasy, 2021).

### Cytotoxicity on Vero Cells

To study the cytotoxic effect of CZME on Vero cells, some concentrations of CZME were separated into the 96-well microplate containing Vero cells (1 × 10^5^ cells/ml) for 24 and 48 h at 37°C with 5% CO_2_. Next, MTT powder (3-(4,5-dimethylthiazol-2-yl)-2,5-diphenyltetrazolium bromide) (5 mg/ml) was added to the tested wells and were against incubated under the same conditions. After adding the stop solution (dimethyl sulfoxide), the optical density (OD) of wells was read at 570 nm using an ELISA reader (LX800; Biotec, USA) (Albalawi et al., 2021). The 50% cytotoxic concentration (CC_50_) was also calculated for Vero cells exposed to CZME.

### Animals

A total of 84 male BALB/c mice were procured from the Animal Breeding Department of the Faculty of Science and Humanities, Shaqra University, Saudi Arabia. The animals were held at a proper temperature (24 ± 1°C), with a 12-h light/dark cycle and absolute humidity of 40–70%. They were fed with adequate water and food *ad libitum*.

### Ethics

The Ethical Committee for Animal Experiments of Almaarefa University, Saudi Arabia (No. IRB07-18052022-46) approved the current work.

### Induction of the Animal Model of *T. gondii* Infection

The induction of latent toxoplasmosis in mice was based on the method described elsewhere (Saadatmand et al., 2021). Briefly, the mice were intraperitoneally inoculated with 500 µl of suspension from the brain having *T. gondii* tissue cysts (20–25 cysts) along with streptomycin and penicillin antibiotics. The infection was confirmed by assessing the anti-*T. gondii* IgG antibody in mice sera by using a modified agglutination test (MAT) kit (Toxo screen DA, Biomérieux, Lyon, France), where the titer of ≥1:20 was considered positive.

### Therapeutic Effects on Mice Infected With Toxoplasmosis

To study the therapeutic effects of CZME on mice infected with toxoplasmosis, one day post-infection, 60 infected mice were accidentally allocated to five groups, containing 12 mice from each group, including:

- 14 days of oral treatment with normal saline.
- 14 days of oral treatment with atovaquone at a dose of 50 mg/kg/day.
- 14 days of oral treatment with CZMO at a dose of 20 mg/kg/day.
- 14 days of oral treatment with CZMO at a dose of 40 mg/kg/day.
- 14 days of oral treatment with CZMO at a dose of 60 mg/kg/day.
- The selection of these doses was based on previous studies and primary experiments (Sayad-Fathi et al., 2020; Atsamo et al., 2021).

### Assessment of Oxidative Stress Factors

One day after 14 days of treatment (on the 15th day post-treatment), through the intraperitoneal injection of sodium pentobarbital, six mice from each group were deeply euthanized to assess the level of oxidative stress factors [liver lipid peroxidation (LPO) and nitric oxide (NO)] and some proinflammatory cytokines. The level of LPO and NO in liver homogenates was examined according to the commercial kits (Abcam, USA), carried out as a malondialdehyde (MDA) colorimetric assay (Albalawi et al., 2022). NO production was also determined in the liver suspension based on the NO colorimetric kit guidelines of the manufacturer (ab65328, Abcam).

### Assessment of Antioxidant Enzymes

We assessed the level of some antioxidant factors, e.g., glutathione peroxidase (GPx), catalase enzyme (CAT), and superoxide dismutase enzyme activity (SOD), based on the guidelines of the manufacturer of the commercial kits (Abcam, USA) as introduced by Weydert et al. (Weydert and Cullen, 2010), Luck (1965), and Sun et al. (1988), respectively.

### Measuring the Proinflammatory Cytokines

The levels of interferon-gamma (IFN-γ), interleukin 1-beta (IL-β), and IL-12 were studied based on the guidelines of the manufacturer of the commercial kits (Abcam, USA).

### Collection of Brain Samples

On the 60th day, using the i.p. inoculation of ketamine xylazine (15 mg/kg) and (100 mg/kg), the mice in the tested groups were deeply anesthetized. Following decapitation of the mice, the whole brain tissue was collected aseptically; the right and left brain hemispheres were used for parasitological and molecular tests, respectively.

### Effects on *T. gondii* Tissue Cysts

The antiparasitic activity of CZMO was evaluated after preparing unstained smears from the brain tissue of the tested mice. In the next step, using light microscopy, the diameter and number of *T. gondii* tissue cysts were calculated (Saadatmand et al., 2021).

### Effects on the Expression Level of Bradyzoite Surface Antigen 1 (BAG1)

The effects of CZEO on the mRNA levels of BAG1 were studied *via* quantitative real-time PCR based on the Minimum Information for Publication of Quantitative Real-Time PCR Experiments (MIQE) guidelines (Sun et al., 1988). Initially, the total RNA of the brain of the mice was extracted based on the protocol of the commercial kit (Qiagen, Hilden, Germany). The extracted total brain RNAs were then reverse-transcribed based on the guidelines of the producer, and the acquired complementary DNA (cDNA) was applied for real-time PCR through SYBR green (Ha et al., 2010). During real-time PCR, the conditions of temperature were an early denaturation at 96°C for 11 min, 40 cycles of amplification [denaturation at 96°C for 10 s, annealing at 57°C for 30 s, and elongation at 72°C for 30 s], and then one cycle at 72°C for 5 min. Finally, the data were analyzed by calculating 2^−ΔΔCT^. **Table 1** displays oligonucleotide sequences used for real-time PCR. All tests were performed in triplicate to guarantee replicability (Azami et al., 2018).

**Table 1:** The oligonucleotide primers applied for real-time PCR.

Amplicon	Primers	Sequence (5’–3’)	Size (bp)
BAG1	F R	AGTCGACAACGGAG CCATCGTTATC ACCTTGATCGTGACACGTAGAACGA	182
β-actin	F R	GTGACGTTGACATCCGTAAAGA GCCGGACTCATCGTACTCC	245

### Statistical Analysis

The experiments were conducted in triplicate. Data analysis was performed on SPSS 22.0. The difference in results among the tested groups was also determined by one-way ANOVA with Turkey’s *potshot* test. P <0.05 was statistically significant.

## Results

### Phytochemical Examination

The *C. zeylanicum* yielded a 7.98% w/v dry methanolic extract. The phytoconstituent tests on CZME revealed alkaloids, terpenoids, flavonoids, tannins, and saponins in CZME.

### Secondary Metabolite *C*ontents

The results revealed that the concentration of phenol and flavonoid phytochemicals was 59.65 ± 2.21 mg GAE/g DW and 34.32 ± 1.36 mg CEA/g DW, respectively.

### GC/MS Analysis of CZME

The GC/MS examination of CZME proved the existence of 13 chemical constituents (**Table 2**). The most abundant constituents were cinnamaldehyde (90.3%), trans-cinnamyl acetate (2.1%), and trans-ethyl p-methoxycinnamate (1.6%), respectively.

**Table 2:** Chemical composition of *C. zeylanicum* methanolic extract determined by GC/MS.

Peak No.	Compound	RI^a^	Area (%)
1.	α-pinene	923	0.8
2.	Camphene	943	0.8
3.	Cis-ocimene	1,031	0.4
4.	Linalool	1,102	0.4
5.	(Z)-3-Phenylacrylaldehyde	1,215	0.3
6.	Cinnamaldehyde	1,262	90.3
7.	*trans*-cinnamyl acetate	1,276	2.1
8.	α-Copaene	1,365	0.6
9.	(E)-Caryophyllene	1,422	0.5
10.	Humulene	1,428	0.1
11.	α-Calacorene	1,547	0.5
12.	6-Phenyl-3,5-hexadien-2-one	1,651	0.7
13.	trans-Ethyl p-methoxycinnamate	1,762	1.6
	Total		99.1

a Retention index.

### *In Vitro* Cell Viability Assay

**Figure 1:**
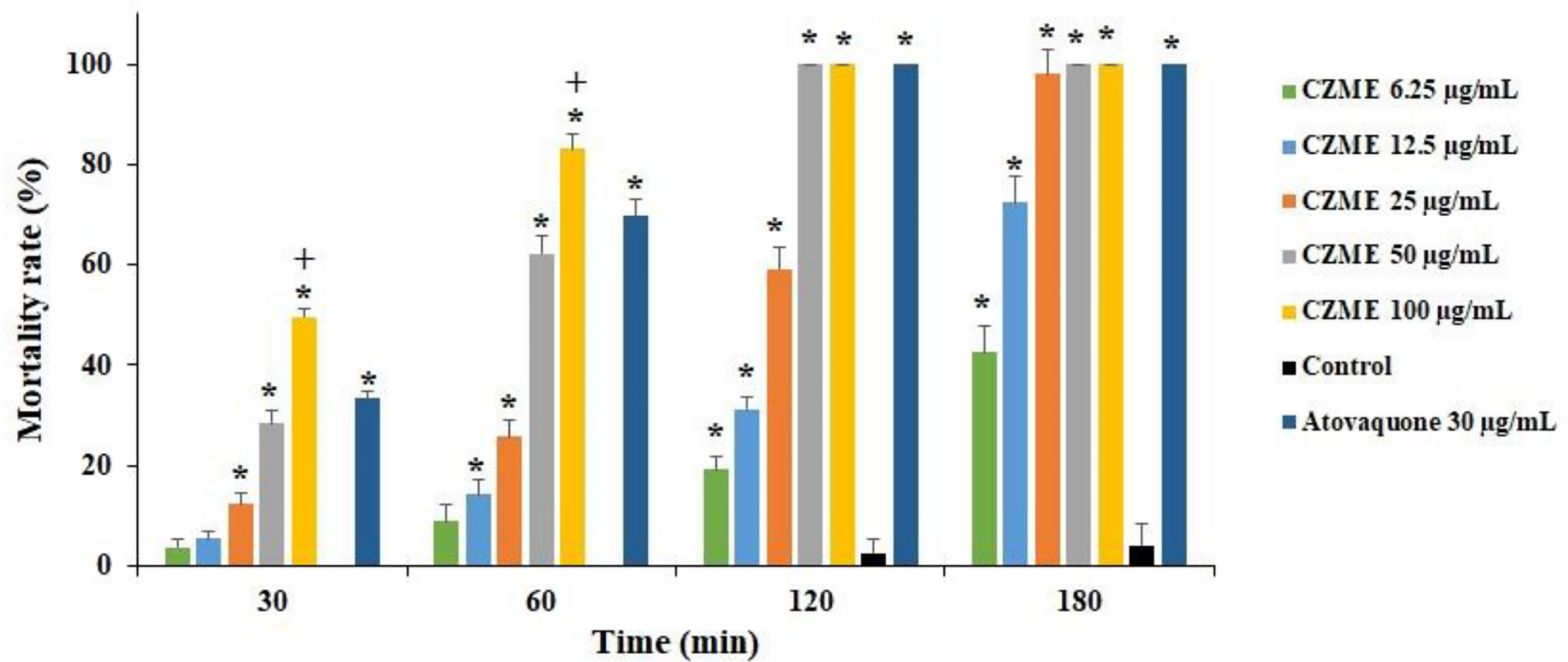
*In vitro* effects of *C. zeylanicum* methanolic extract (CZME) against *T. gondii* tachyzoites after different incubation times. Results are displayed as the mean ± SD (n = 3). *P < 0.001 represents a significant difference in comparison with the control group; +p < 0.001 represents a significant difference in comparison with the atovaquone.

**Figure 1** shows the effect of various concentrations of CZME on the viability of *T. gondii* tachyzoites. Based on the results of the MTT assay, after 0.5–3 h of incubation of *T. gondii* tachyzoites with CZME (25, 50, and 100 µg/ml), the mortality rate of parasites was significantly increased in a dose- and time-dependent response compared with the control group. However, CZME at a concentration of 100 and 50 µg/ml eliminated parasites after 2 and 3 h of exposure. Additionally, using atovaquone as the control drug caused a significant reduction in the mortality rate of parasites.

### Determining the Rate of Infection and Intracellular Parasites

**Figure 2:**
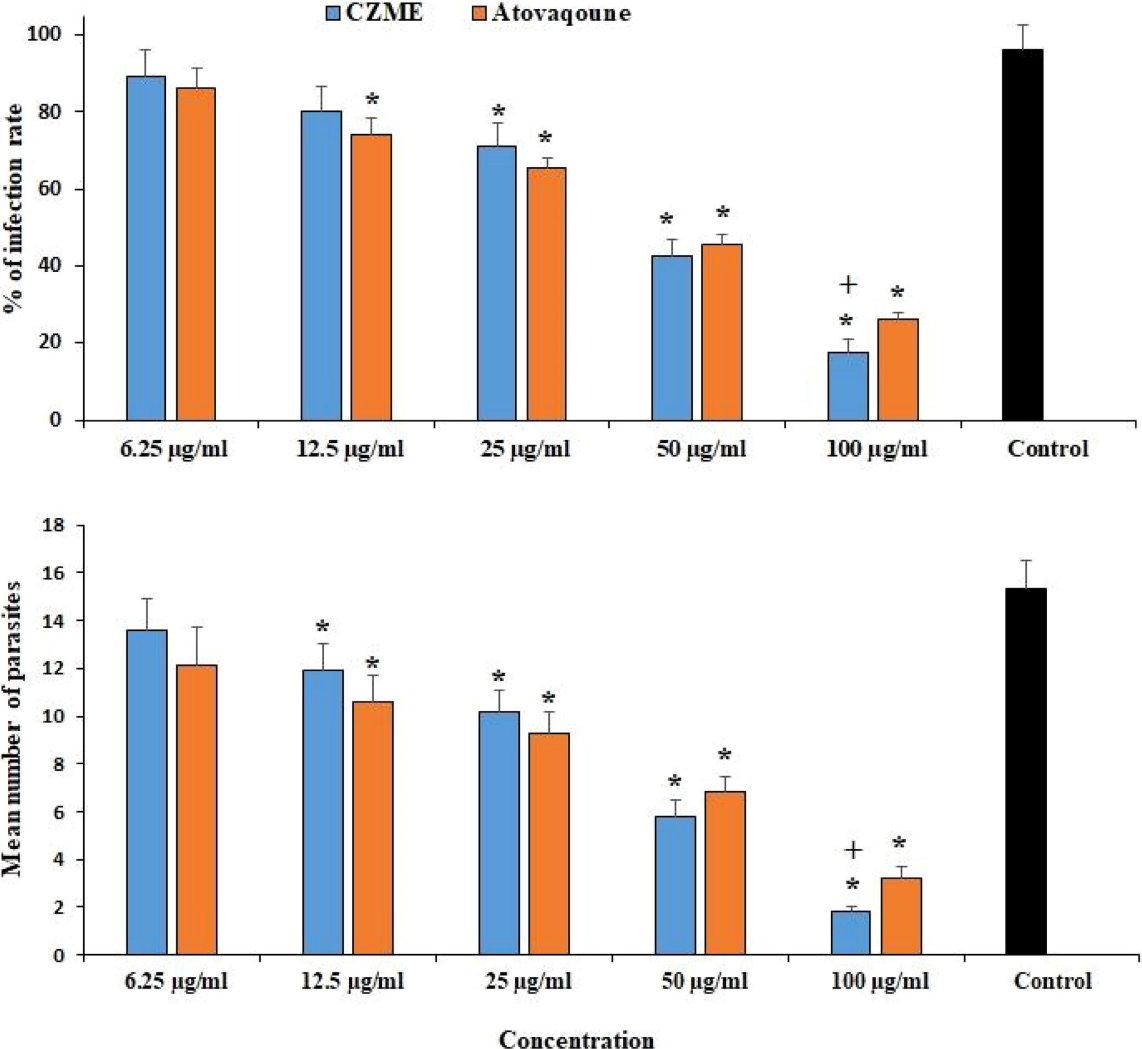
Effect of *C. zeylanicum* methanolic extract (CZME) on the infection rate of the Vero cells and mean number of intracellular parasites after 3 h incubation. Mean ± SD (n = 3). *p <0.001 compared with the control group; +p <0.05 compared with atovaquone.

The maximum inhibitory activity of CZME was detected at 100 µg/ml, where it considerably reduced the infection rate (p <0.001) by 17.6%. CZME at doses of 25 and 50 µg/ml also decreased (p <0.05) the infection rate by 71.3 and 42.4%, respectively. According to the microscopic examinations, the mean number of intracellular tachyzoites significantly declined (p <0.001) after the exposure of infected cells to CZME (**Figure 2**).

### Cytotoxicity on Vero Cells

The MTT assay results revealed that CZME had no significant cytotoxicity against Vero cells, with a CC_50_ value of 169.5 ± 5.66 μg/ml and 146.3 ± 6.41 μg/ml for CZME and atovaquone, respectively.

### Therapeutic Effects on Mice Infected With Toxoplasmosis

As shown in **Table 3**, the mean number of tissue cysts considerably declined (p <0.001) in a dose-dependent response compared to the control mice (C2); in the infected mice receiving CZME at a dose of 60 mg/kg, only 6.0 tissue cysts were observed. Subsequently, the mean diameter of cysts was also significantly reduced (p <0.001) by 33.3, 51.7, and 78.1% after treating infected mice with CZME at doses of 20, 40, and 60 mg/kg/day, respectively. Additionally, atovaquone as the control drug significantly decreased the mean number and diameter of tissue cysts by 94.9 and 66.1%, respectively.

**Table 3:** The mean number and the mean diameter of the brain tissue cysts followed by pre-treatment of infected mice with *C. zeylanicum* methanolic extract (CZME) for two weeks.

Group	No. of tissue cyst (Mean ± SD)	% of reduction	Diameter of tissue cysts (Mean ± SD)	% of reduction
CZME 20 mg/kg	76.3 ± 4.16*	65.2	54.2 ± 5.21*	33.3
CZME 40 mg/kg	34.6 ± 1.78*	84.2	39.3 ± 4.24*	51.7
CZME 60 mg/kg	6.0 ± 0.0*	97.3	17.8 ± 2.23*	78.1
Control	219.3 ± 6.7	–	81.3 ± 9.14	–
Atovaqoune (50 mg/kg)	11.2 ± 2.0*	94.9	27.6 ± 2.19*	66.1

*p < 0.001.

### Assessing the Oxidant/Antioxidant Factors

**Figure 3:**
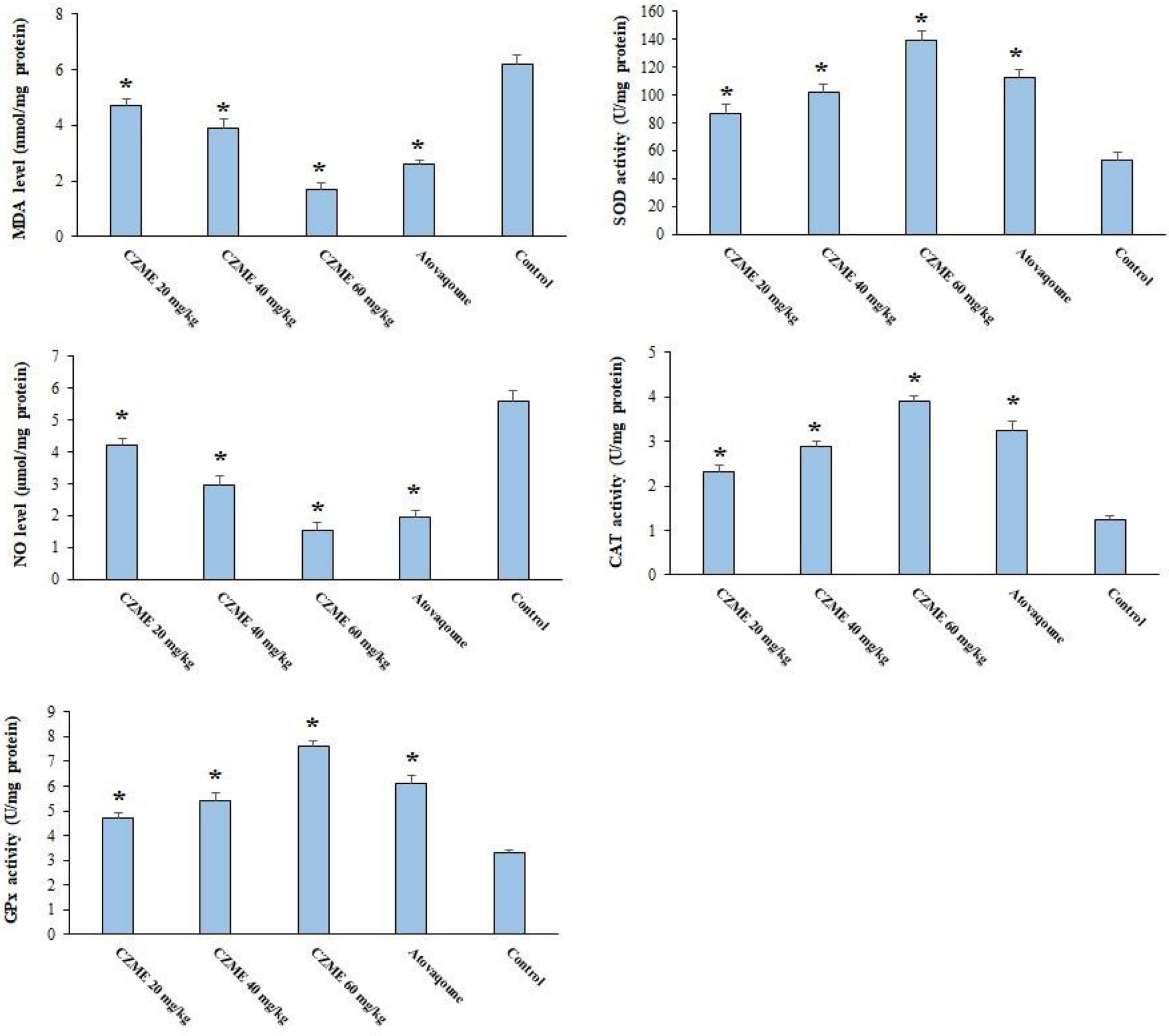
Effect of *C. zeylanicum* methanolic extract (CZME) on the malondialdehyde (MDA), nitric oxide (NO), glutathione peroxidase (GPx), superoxide dismutase enzyme activity (SOD), and catalase enzyme (CAT) in infected mice in comparison with control groups. Mean ± SD (n = 8). *p < 0.001.

As depicted in **Figure 3**, in infected mice treated with normal saline, the amount of MDA and NO was increased, while they displayed a decreased level of GPx, CAT, and SOD as antioxidant enzymes. Based on the results, after treatment with CZME, the level of LPO and NO was noticeably (*p <*0.05) reduced; in addition, mice showed a significant elevation (*p <*0.05) in the value of GPx, CAT, and SOD.

### Determining the Proinflammatory Cytokines

**Figure 4:**
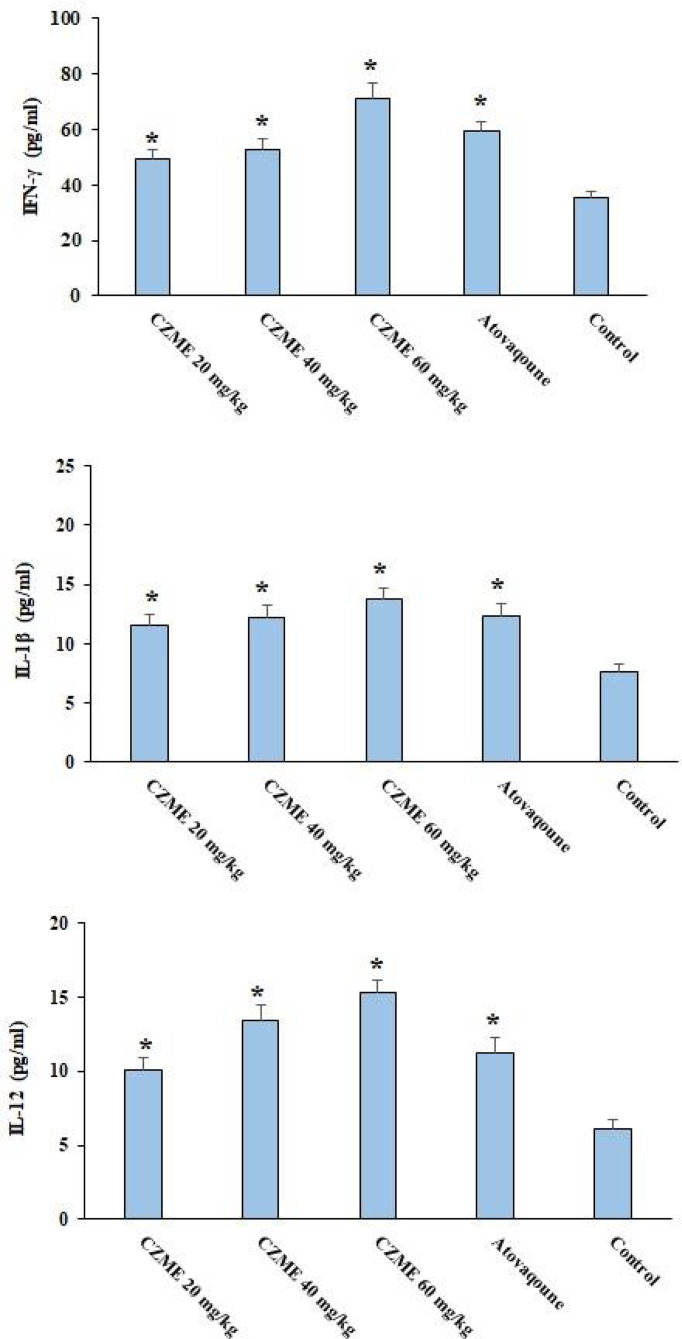
Effect of *C. zeylanicum* methanolic extract (CZME) on the level of IFN-γ, IL-1β, and IL-12 in the infected mice in comparison with the control groups. Mean ± SD. *p < 0.05.

**Figure 4** shows the levels of some proinflammatory cytokines. The finding revealed that after treating the infected mice with CZME, the levels of IFN-γ, IL-1β, and IL-12 were considerably elevated in a dose-dependent response (p <0.05).

### Effects on the Expression Level of BAG1

**Figure 5:**
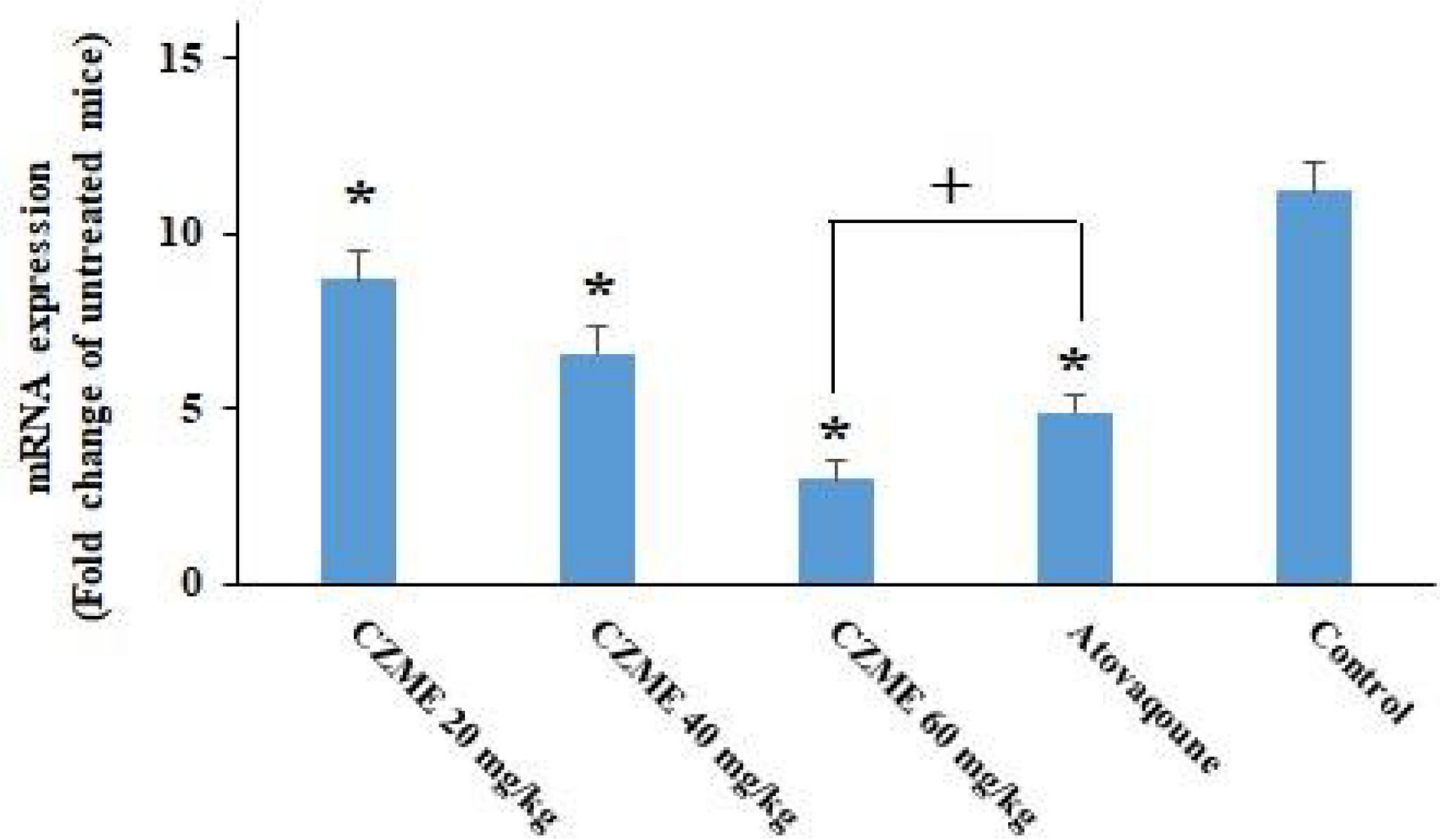
The effects of *C. zeylanicum* methanolic extract (CZME) on the expression level of bradyzoite surface antigen 1 (BAG1) in infected mice. But treatment of infected mice with CZME results in a significant (p < 0.001) downregulation of BAG1 in a dose-dependent response (p < 0.05). Mean ± SD. *p < 0.001 compared with the control group; +p < 0.05 compared with atovaqoune.

Based on the findings of real-time PCR (**Figure 5**), although the expression level of BAG1 was significantly upregulated in the infected mice, the treatment of the infected mice with CZME resulted in a significant (p <0.001) downregulation of BAG1 with increasing doses of CZME (p <0.05). The results also revealed that CZME at a dose of 60 mg/kg displayed a significant (p <0.05) downregulation in BAG1 compared with atovaquone.

### Toxicity Effects of CZME on Liver and Kidney Function

**Figure 6:**
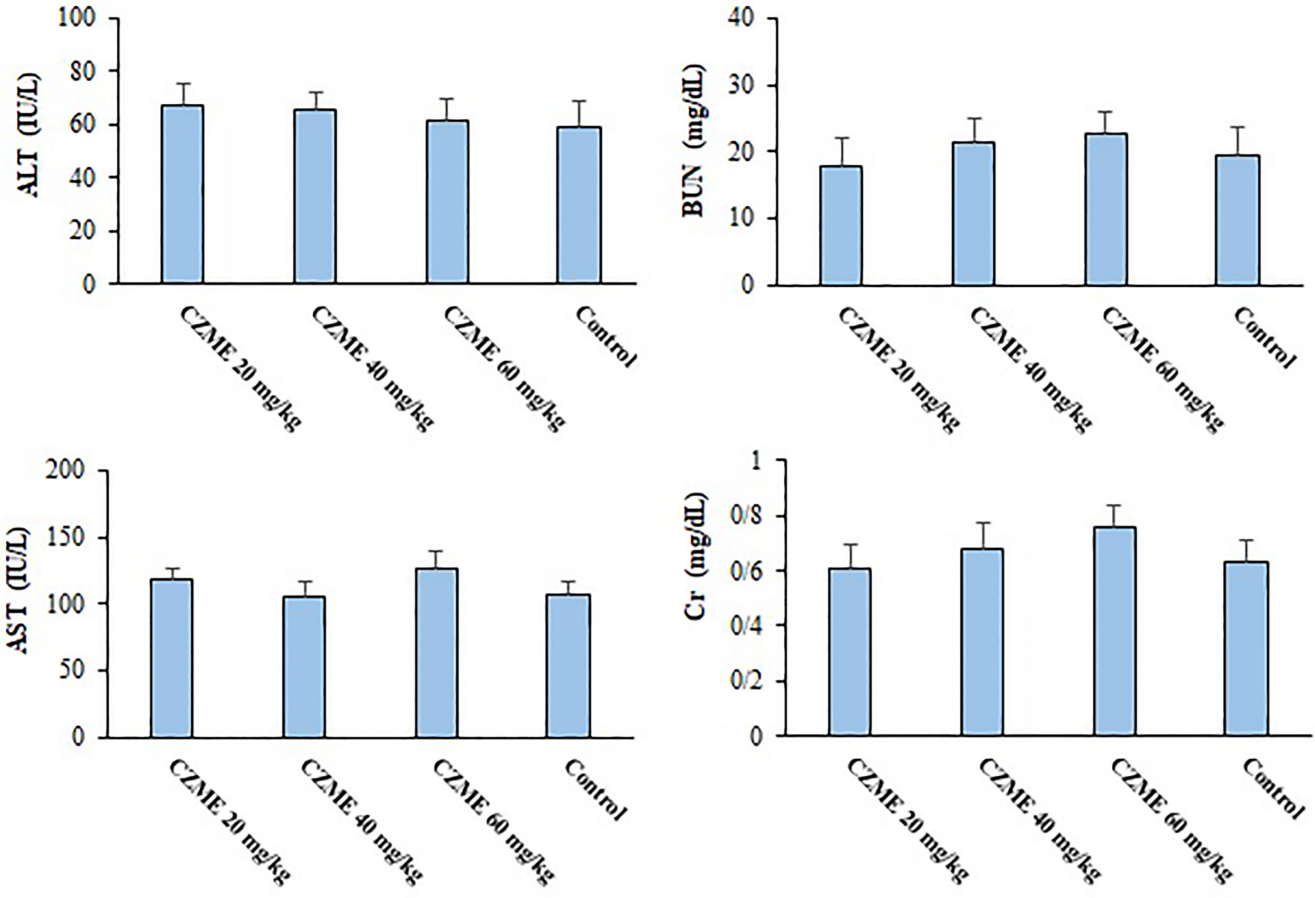
The effects of *C. zeylanicum* methanolic extract (CZME) on the level of aspartate aminotransferase (AST), alanine aminotransferase (ALT), Blood Urea Nitrogen (BUN) and Creatinine (Cr) in the healthy mice. Mean ± SD.

The results indicated that, followed by 14 days of treatment of healthy mice with CZME at the doses of 20, 40, and 60 mg/kg/day, no significant alteration was reported in the serum levels of liver and kidney enzymes, e.g., ALT, AST, BUN, and Cr, when compared to the control mice (**Figure 6**).

## Discussion

According to recent reports, the existing synthetic drugs (e.g., pyrimethamine, sulfadiazine, atovaquone, and spiramycin) for treatment and prevention of toxoplasmosis are associated with severe side effects such as increased risk of suppression of bone marrow function, hematologic toxicity, teratogenic effects, and limited renal complications (Alday and Doggett, 2017; Lapinskas and Ben-Harari, 2019). Despite significant advances in pharmacological and safety research, there is still no ideal and effective medication for toxoplasmosis; hence, the preparation of a new drug that has properties such as effective in penetrating the placenta, non-toxicity, and anti-parasitic effects on all stages of the parasite, especially the cystic form, seems necessary.

There has been a rising trend of studies on the efficiency of herbal products on various infections, including parasitic ones (Thillaivanan and Samraj, 2014). Recently, various *in vitro, in vivo*, and clinical trials laboratory studies reported that some plant extracts and derivatives have potent antiprotozoal effects (Bahmani et al., 2014). Several experimental investigations in various parts of the world have examined the anti-*Toxoplasma* effects of plant extracts and their derivatives against various strains of *T. gonsii* (Wei et al., 2015; Cheraghipour et al., 2021). However, due to some limitations (e.g., using herbs in a specific area that is inaccessible to all, using different strains of the parasite, the uncertainty of their toxicity, contentment with *in vitro* studies, and failure to study the mechanisms of action), their results have not yet been finalized and are unreliable (Cheraghipour et al., 2021). For these reasons, researchers must evaluate a new plant with potential biological activity, low toxicity, and high availability. The current study is intended to evaluate the *in vitro*, therapeutic, and toxicity effects of *C. zeylanicum* methanolic extract against *T. gondii* infection.

Based on the *in vitro* results, after 0.5, 1, 2, and 3 h of incubation of *T. gondii tachyzoites* with different concentrations of CZME, the mortality rate of parasites was significantly increased in a dose and time-dependent response. Furthermore, the inhibitory activity of CZME on the infection rate in the infected cells was detected, especially at a concentration of 100 µg/ml, where it was considerably reduced by 17.6%. Microscopic examinations also indicated that the mean number of intracellular tachyzoites significantly declined after exposure to CZME. Considering the therapeutic effects of CZME on mice infected with toxoplasmosis, the findings showed that CZME therapy significantly declined the mean number and the mean diameter of *T. gondii* tissue cysts in infected mice. In addition to microscopic examinations to study the effect of CZME on the number and size of *T. gondii* tissue cysts in mice, the expression of the BAG1 gene has been measured in *T. gondii* mice treated with CZME using real-time PCR. Although the expression level of BAG1 was significantly upregulated in infected mice, the treatment of infected mice with CZME resulted in a significant (p <0.001) downregulation of BAG1 in a dose-dependent response. Even though these findings showed the promising *in vitro* effects of CZME on *T. gondii*, more studies, especially regarding the mechanism of action of CZME, must be conducted to confirm these results. These results showed the promising *in vitro* and *in vivo* effects of CZME on *T. gondii* infection; still, more studies, especially regarding the mechanism of action of CZME, are warranted to confirm these findings.

At present, the antimicrobial activities of *C. zeylanicum* against various bacterial (e.g., *Bacillus* spp., *Mycobacterium* spp., *Staphylococcus* spp., *Escherichia coli*), fungal (*Aspergillus* spp., *Candida* spp.), and viral (e.g., human immunodeficiency virus, influenza H1N1, herpes simplex) pathogens have been proven in numerous *in vitro* and *in vivo* investigations (Ranasinghe et al., 2013; Brochot et al., 2017). Concerning the anti-parasitic effects of *C. zeylanicum*, Fabbri et al. have demonstrated promising effects of *C. zeylanicum* and the main compound cinnamaldehyde (25–200 μg/ml) on protoscoleces and larval stages of *Echinococcus granulosus* (Fabbri et al., 2020). Salama et al. (2021) have reported that treating mice with *Trichinella spiralis* infection with *C. zeylanicum* displayed a significant reduction in the viability of larvae and adult worms of *T. spiralis* (Salama et al., 2021). In an investigation conducted by Rakhshandehroo et al. (2016), the findings showed that *C. zeylanicum* at concentrations of 50–125 mg/ml displayed potent anti-parasitic effects on infective larvae of *Parascaris equorum* after 10–40 min of incubation (Rakhshandehroo et al., 2016). Jorjani et al. (2017) have also revealed the relevant antileishmanial effects of *C. zeylanicum* at 31.25–1,000 μg/ml against promastigote forms of *Leishania major* (Jorjani et al., 2017). In the study conducted by Ghanbariasad et al. (2021), *C. zeylanicum* displayed potent antileishmanial activity against *L. major* and *L. tropica* with 50% inhibitory concentrations of 16.53 and 7.56 μg/ml, respectively (Ghanbariasad et al., 2021).

Similar to most previous surveys (Ranasinghe et al., 2013; Rahayu et al., 2021), we reported the presence of flavonoids, tannins, alkaloids, terpenoids, and saponins in CZME and cinnamaldehyde, trans-cinnamyl acetate, and trans-ethyl p-methoxycinnamate as the most abundant constituents of CZME by GC/MS analysis. Nevertheless, it has been supported several times that the chemical composition of plants may be influenced by factors such as the place and time of harvest, the part of the plant used for analysis, and the method of essential oil extraction (Masoumi-Ardakani et al., 2016; Shaapan et al., 2021). Cinnamaldehyde is the main compound of CZME and has a broad range of medicinal and pharmacological applications such as anticancer, cardio-protection, anti-inflammatory, antibacterial, antifungal, and anti-parasitic activities (Chen et al., 2017; Doyle and Stephens, 2019). As for the antimicrobial mechanisms of action of cinnamaldehyde, investigations showed that this compound displays its antimicrobial effects through multiple mechanisms, e.g., by disrupting the metabolism of some vital compounds such as proteins, lipids, and carbohydrates, by distraction of the microbial cell membrane, and by disrupting energy production (Gill and Holley, 2004; Nowotarska et al., 2017). Therefore, it can be deduced that the anti-*Toxoplasma* effect of CZME is due to the presence of this compound in this plant.

LPO is well-known as an indicator of oxidative stress, which leads to cell membrane destruction followed by the discharge of indicator enzymes of hepatotoxicity (Souza et al., 2017). Toxoplasmosis may tissue damage, especially in the acute phase, by raising lipid levels and subsequently increasing the production of free radicals (Souza et al., 2017). Our results in this experimental study showed that after CZME therapy of infected mice, the level of LPO and NO, the oxidative stress markers, was noticeably reduced. In contrast, a significant rise (p <0.05) in the level of antioxidant markers such as GPx, CAT, and SOD was reported. In line with our results, several *in vivo* and clinical studies have proven the antioxidant activity of *C. zeylanicum* through increasing antioxidant markers such as GPx, CAT, and SOD (Roussel et al., 2009; Ghosh et al., 2015). The experimental findings revealed that CZME probably improves the symptoms of toxoplasmosis through its antioxidant and anti-inflammatory effects. However, further studies are needed to substantiate this claim.

A vital mechanism that plays an essential role in the prevention and even control of toxoplasmosis infection is the reinforcement of the immune system, especially cellular immunity, by increasing some pro-inflammatory factors and mediators such as interleukin 12 (IL-12) released by immune cells, e.g. macrophages and dendritic cells in reaction to Toll-like receptor (TLR) identification of molecular structures mostly preserved among microbial strains (Melo et al., 2011). Through activation of NK and T cells, IL-12 results in the production of IFN-γ (Gorfu et al., 2014). The results of our investigation indicated that after CZME therapy of infected mice, the level of proinflammatory cytokines of IFN-γ, IL-1β, and IL-12 were markedly elevated in a dose-dependent response; it was also demonstrated that the treatment with CZME may increase the host’s survival and control infection by improving the immune system as it has an indirect effect against toxoplasmosis. However, more supplementary immunological studies are warranted to determine the effects of this extract on strengthening the immune system and the subsequent control of toxoplasmosis.

The results also revealed that CZME has no significant cytotoxicity against Vero cells, with a CC_50_ value of 169.5 μg/ml and 146.3 μg/ml for CZME and atovaquone, respectively. In line with our findings, Husaina et al. (2019) have reported that *C. zeylanicum* ethanolic extract displays no significant activity on Vero cells even at a dose of 100 µg/ml (Husain et al., 2018). Furthermore, Ahmad et al. (2015) have found that oral administration of the aqueous extract of *C. zeylanicum* at doses of 0.1–2 g/kg for 14 days has no considerable toxicity on body weight, food/water consumption, mortality, liver and kidney weight, and hematological factors in female Sprague–Dawley rats (Ahmad et al., 2015). These findings, combined with the results of the current study, show that CZME at the *in vitro* and *in vivo* concentrations used in this study did not have any significant cytotoxicity or histological toxicity. Still, further studies are needed to confirm this conclusion.

## Conclusion

The results of this experimental study confirmed the promising therapeutic effects of CZME chronic toxoplasmosis in mice as it significantly controlled infection by reducing the number and size of tissue cysts, reducing oxidative tissue stress and boosting pro-inflammatory cytokines. At the tested doses, it had no significant toxicity to vital animal tissues. Nevertheless, further investigations must confirm these results, elucidate the precise mechanisms, and show the effectiveness of this substance in human volunteers. Moreover, we intend to continue the study of the *in vitro* and *in vivo* anti-*Toxoplasma* effects of cinnamaldehyde as the main constituent of CZME, which may be responsible for the most biological activity of the plant.

## Data Availability Statement

The original contributions presented in the study are included in the article/supplementary material. Further inquiries can be directed to the corresponding author.

## Ethics Statement

The animal study was reviewed and approved by Almaarefa University, Saudi Arabia (No. IRB07-18052022-46).

## Author Contributions

AA: Study design, Methodology, and Writting manuscript and Critical review. HA: Supervisor, Writting manuscript, and Methodology. All authors listed have made a substantial, direct, and intellectual contribution to the work and approved it for publication.

## Conflict of Interest

The authors declare that the research was conducted in the absence of any commercial or financial relationships that could be construed as a potential conflict of interest.

## Publisher’s Note

All claims expressed in this article are solely those of the authors and do not necessarily represent those of their affiliated organizations, or those of the publisher, the editors and the reviewers. Any product that may be evaluated in this article, or claim that may be made by its manufacturer, is not guaranteed or endorsed by the publisher.
